# Supplementary material for: Effects of Nicotine on the Thermodynamics and Phase Coexistence of Pulmonary Surfactant Model Membranes
Source: Membranes (Basel). 2024 Dec 11;14(12):267. doi: 10.3390/membranes14120267 (PMC11678188; doi:10.3390/membranes14120267)
Supplement: Supplementary file 1 [file membranes-14-00267-s001.zip › membranes-3329254-supplementary.pdf]

Supplementary Material

Figure S1: Effect of nicotine on the main phase transition temperature of DPPC multilamellar vesicles. Data is represented by the change of the DPPC main phase transition temperature in the absence ($T_{m,0}$) and presence (T_m) of nicotine, $(\Delta T_m/T_m = (T_m - T_{m,0})/T_m)$, as a function of nicotine concentration. The analysis was based on the binding scheme proposed by Basso et al [1], which took into account the different binding constants of the drug to the gel and fluid lipid phases. The model states that the relative change in the phospholipid phase transition temperature ($\Delta T_m/T_m$) caused by drug binding is $\Delta T_m/T_m = -(RT_{m,0}/\Delta H_0)\ln[(1 + C_F[L]_{Tot})/(1 + C_G[L]_{Tot})]$, where ΔH_0 is the enthalpy change of pure DPPC, $[L]_{Tot}$ represents the total ligand concentration added into the sample solution, and C_F and C_G are fitting parameters that depend both on the partition constant of nicotine to the membrane and on the binding constants of the drug to the membrane gel (K_G) and fluid (K_F) phases. From the fitting, one can obtain the ratio of the binding constants as $K_F/K_G = C_F/C_G$. For nicotine in DPPC, K_F/K_G was determined as 1.7 ± 0.3.

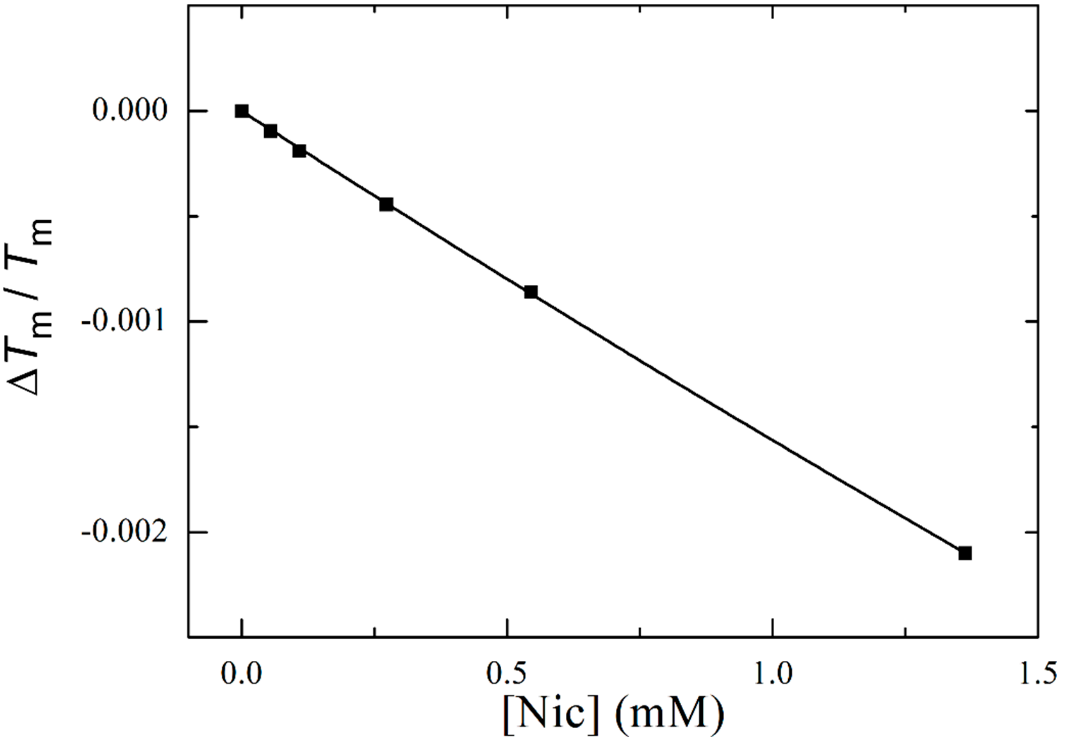

Figure S2: Nicotine structure and molecular abundance of charged states. (A) Molecular structure of nicotine along with the pKa values of the ionizable nitrogen moieties. **(B)** Molecular abundance of the neutral (NTN), monoprotonated (NTH), and diprotonated (NT2H) species of nicotine as a function of the pH. The distribution of the molecular species was modeled according to equations $pH = pKa_1 - \log[molNic/molNic^{H^+}]$ and $pH = pKa_2 - \log[molNic^{H^+}/molNic^{2H^+}]$ [2].

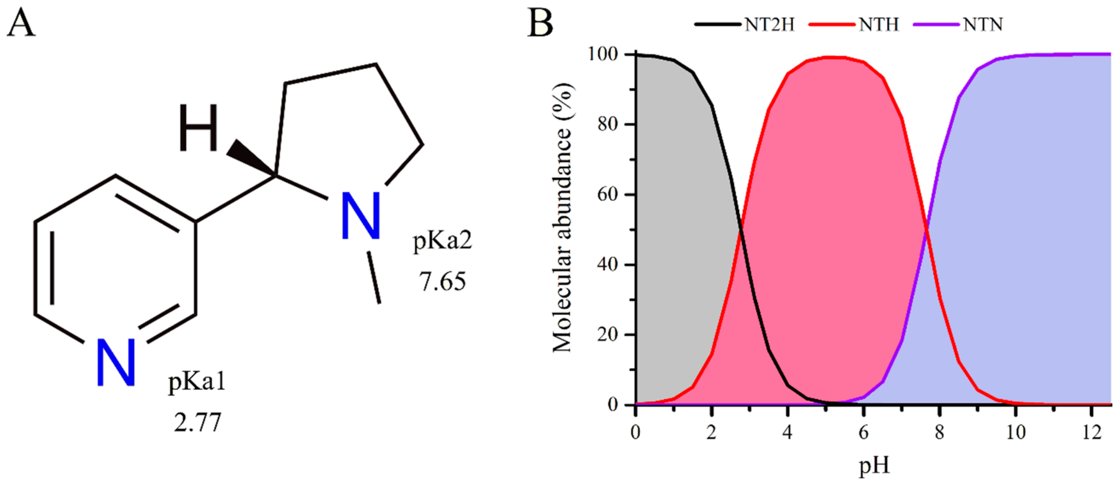

Figure S3: Analysis of the 5-PCSL ESR spectra in DPPC using parameters defined on the spectra. (A) Temperature dependence of the maximum hyperfine splitting ($2A_{max}$) in the absence and presence of 10 mol% of nicotine. The higher the $2A_{max}$ the more packed is the bilayer. **(B)** Temperature dependence of the linewidth of the middle line (ΔH_0) in the absence and presence of 10 mol% of nicotine. The narrower the ΔH_0 the higher the mobility of the spin label. The inset is a magnification of the 37 ºC-to-44 ºC interval.

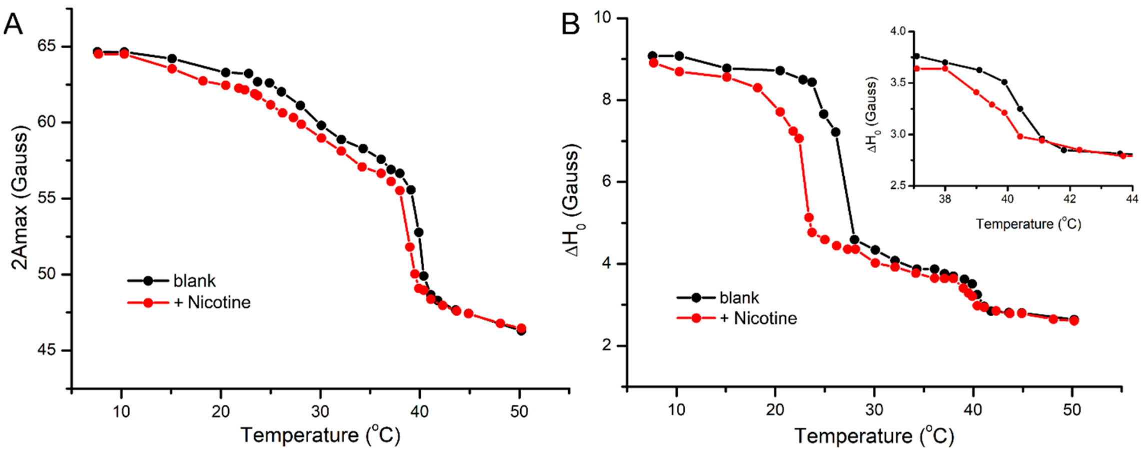

Figure S4: Two-component spectra in the gel phase of DPPC. ESR spectra of 16-PCSL, 16-SASL, and 16-MESL in DPPC multilamellar vesicles obtained at 34 ºC. All spectra display a “shoulder” in the low-field and high-field lines, as indicated by the arrows.

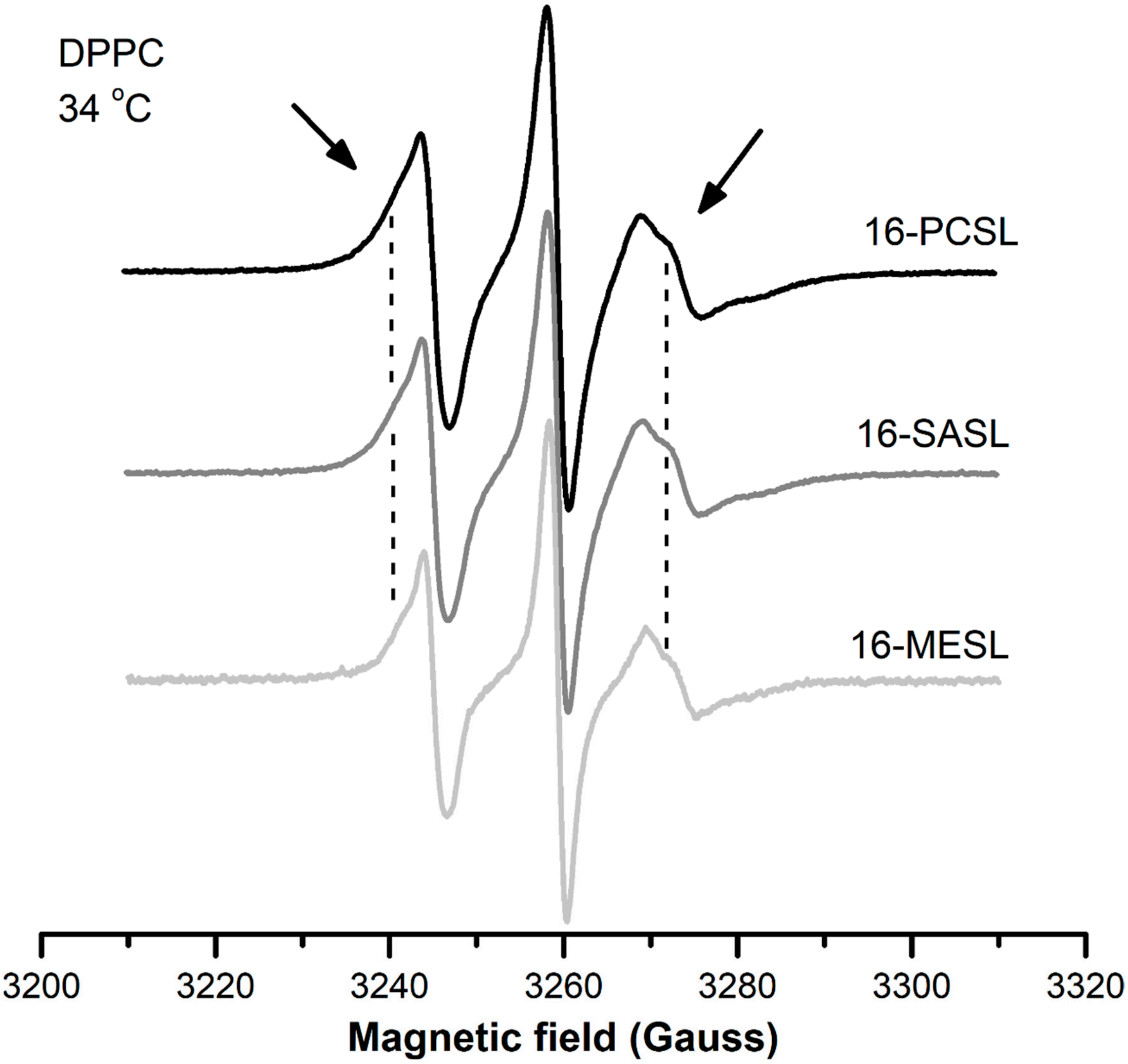

Figure S5: Experimental (black) and best-fit (red) ESR spectra of 16-PCSL in pure DPPC (**A** and **D**) and in DPPC/nicotine lipid vesicles at two concentrations: 2 mol% (**B** and **E**) and 10 mol% (**C** and **F**). Only representative spectra at selected temperatures are illustrated. The spectra in panels A, B and C were simulated with only one component, whereas those in panels D, E and F were fitted with two components (green and blue). Arrows point to the second component in the phase coexistence region.

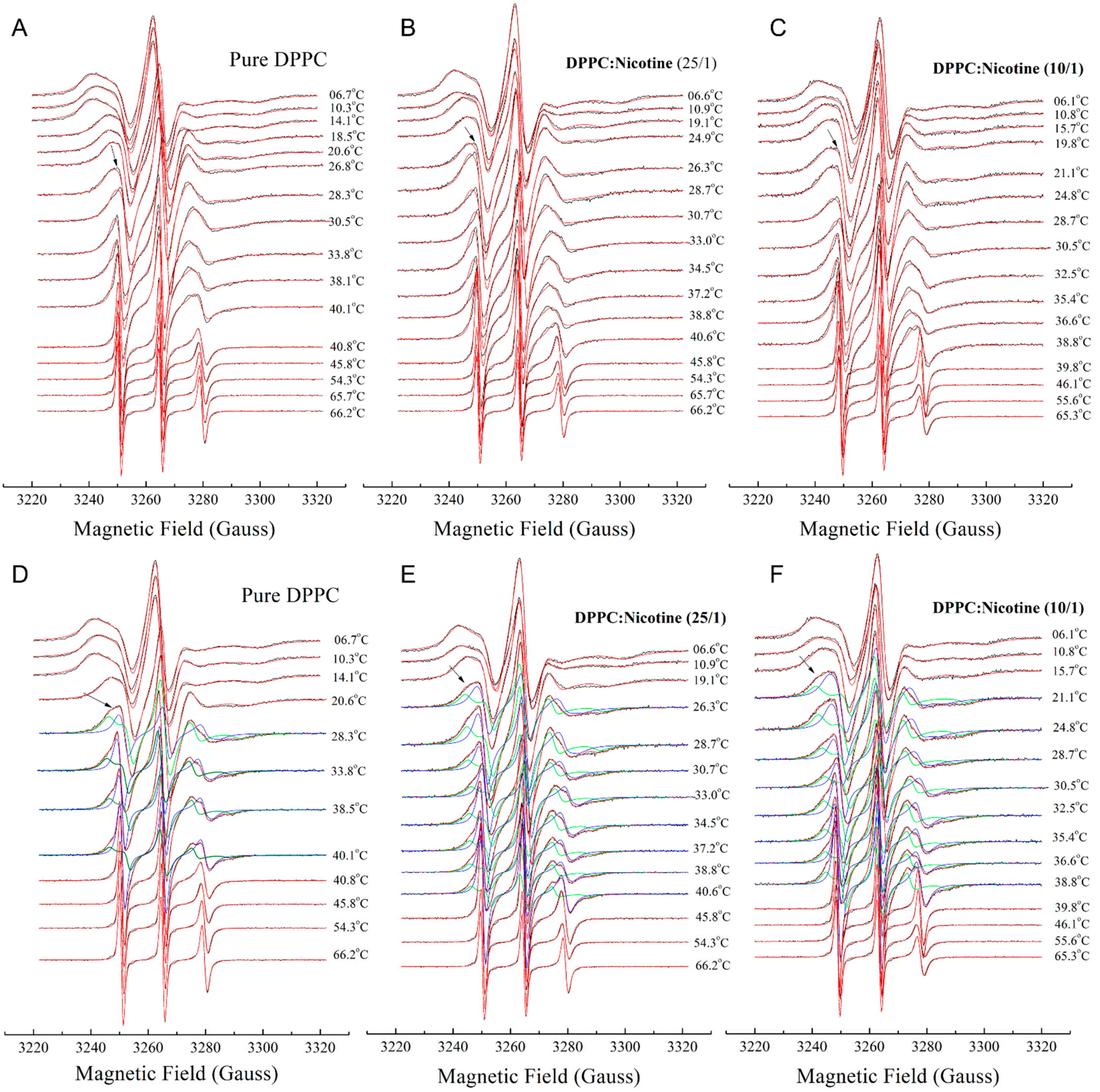

Figure S6: Effect of nicotine on the structural dynamics of the lipid headgroups. ESR spectra of DPPTC in (A) DPPC and (B) DPPC/POPC/POPG bilayers in the absence (black) and presence (red) of 10 mol% of nicotine. The spectra were obtained at the indicated temperatures and using a pH 7.4 buffer for DPPC and pH 7.4 and pH 5.0 buffers for the ternary lipid mixture.

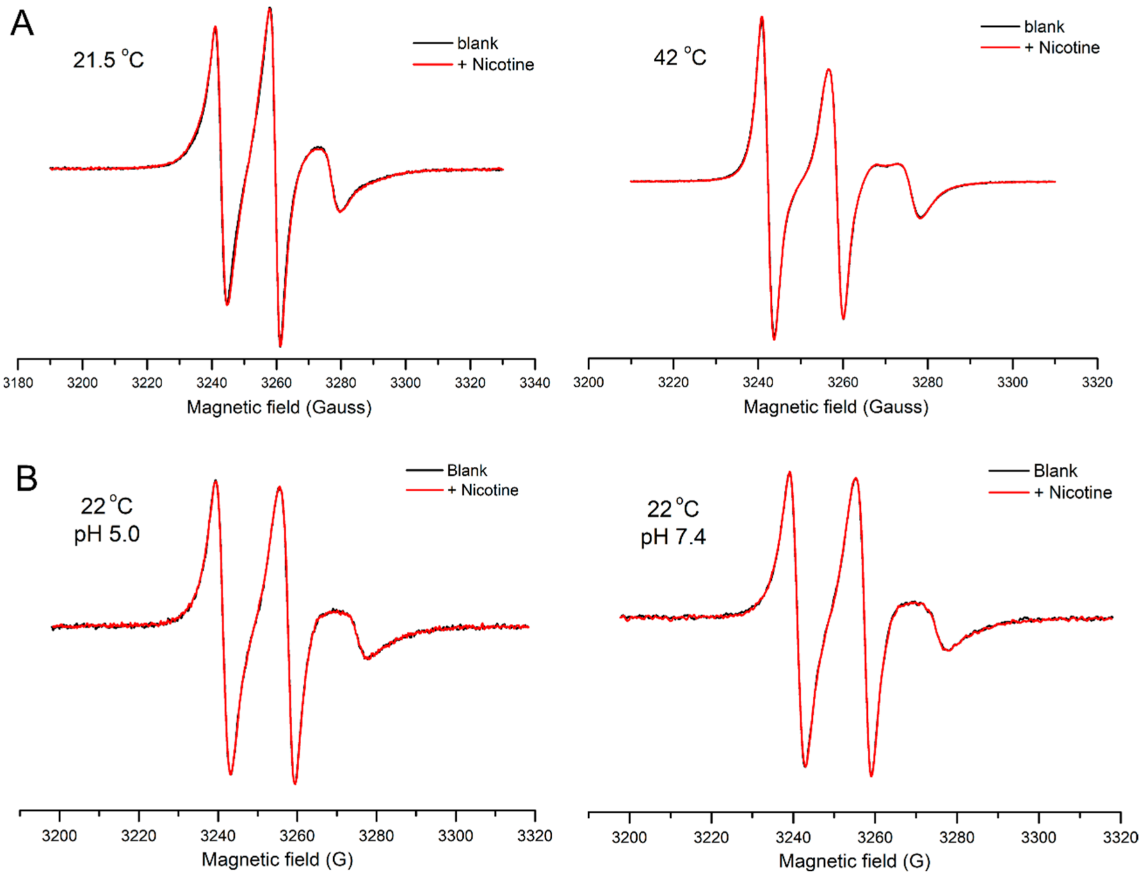

Figure S7: Entropy-enthalpy compensation of the nicotine-free and nicotine-containing pulmonary surfactant membrane models. (A) TΔS x ΔH plots for pure DPPC (full squares) and DPPC/nicotine vesicles at two drug concentrations: 4 mol% (full triangles) and 10 mol% (empty diamonds). (B) TΔS x ΔH plots for DPPC/POPC/POPG (full squares) and nicotine-enriched vesicles at different drug concentrations: 2 mol% (empty circles), 4 mol% (full triangles) and 10 mol% (empty diamonds).

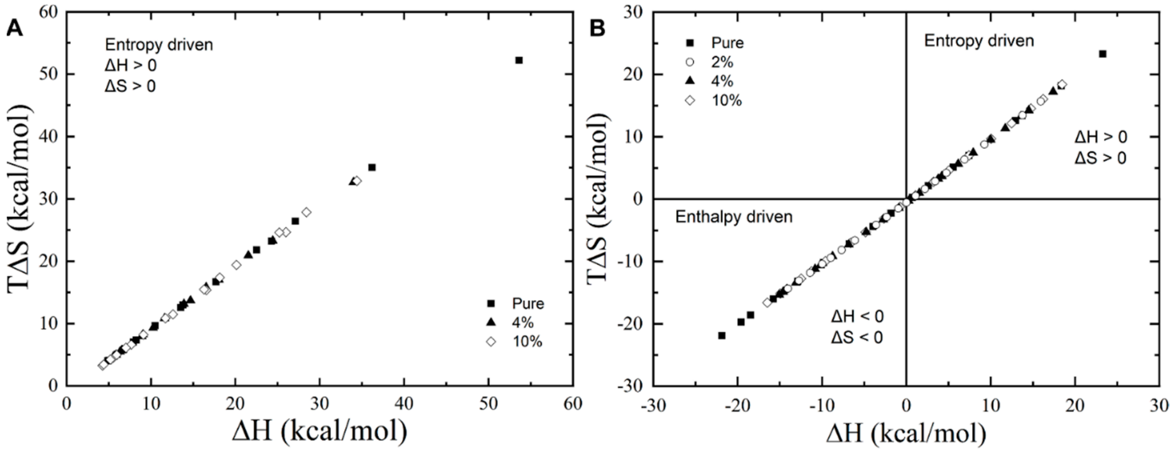

Figure S8. Membrane location of nicotine. MD snapshots showing the location and topology of neutral and monoprotonated nicotine molecules. Panels A and B depict the results for DPPC at 37 °C, panels C and D at 45 °C, while panels E and F represent the data for the lung surfactant model membrane (DPPC/POPC/POPG) at 37 °C. The numbers 1, 3, and 13 following the designation of each system on the top of the panels refer to the number of nicotine molecules in the system. Nicotine molecules are shown in cyan, with nitrogen atoms in blue; water molecules are shown in red, and the lipid bilayer is displayed with phospholipid phosphate atoms in orange spheres and tails in gray.

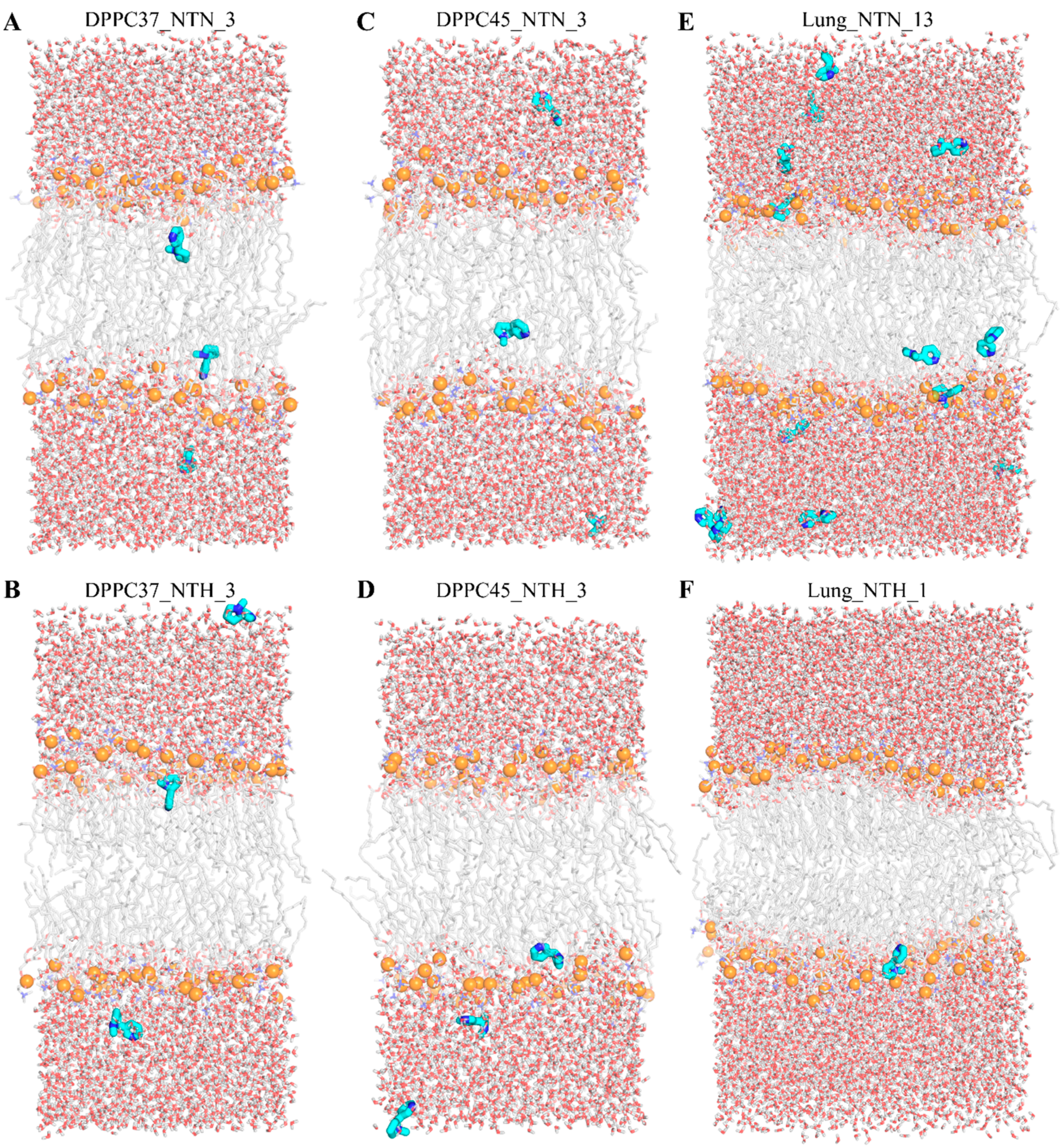

Figure S9: Time-dependent contacts, in percentage, between the protonated and neutral nicotine species with the other molecules (water, lipids, and nicotine) at different concentrations for DPPC at 37 °C. The contact is considered when the distance between any atom (including hydrogen) of two molecules is less than 3 Å. The numbers 1, 3, and 7 following the designation of each system on the top of the panels refer to the number of nicotine molecules in the system.

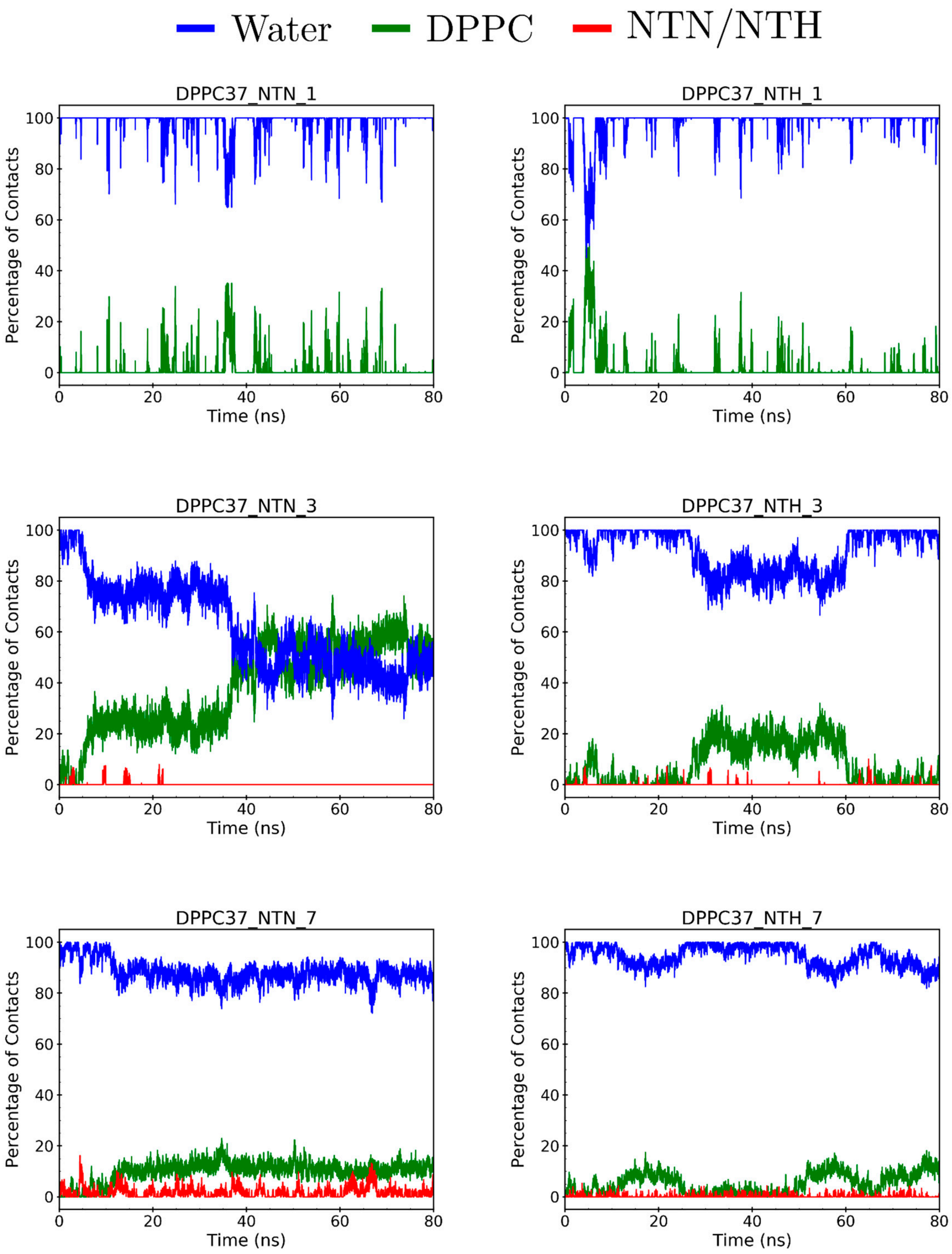

Figure S10: Time-dependent contacts, in percentage, between the protonated and neutral nicotine species with the other molecules (water, lipids, and nicotine) at different concentrations for DPPC at 45 °C. The contact is considered when the distance between any atom (including hydrogen) of two molecules is less than 3 Å. The numbers 1, 3, and 7 following the designation of each system on the top of the panels refer to the number of nicotine molecules in the system.

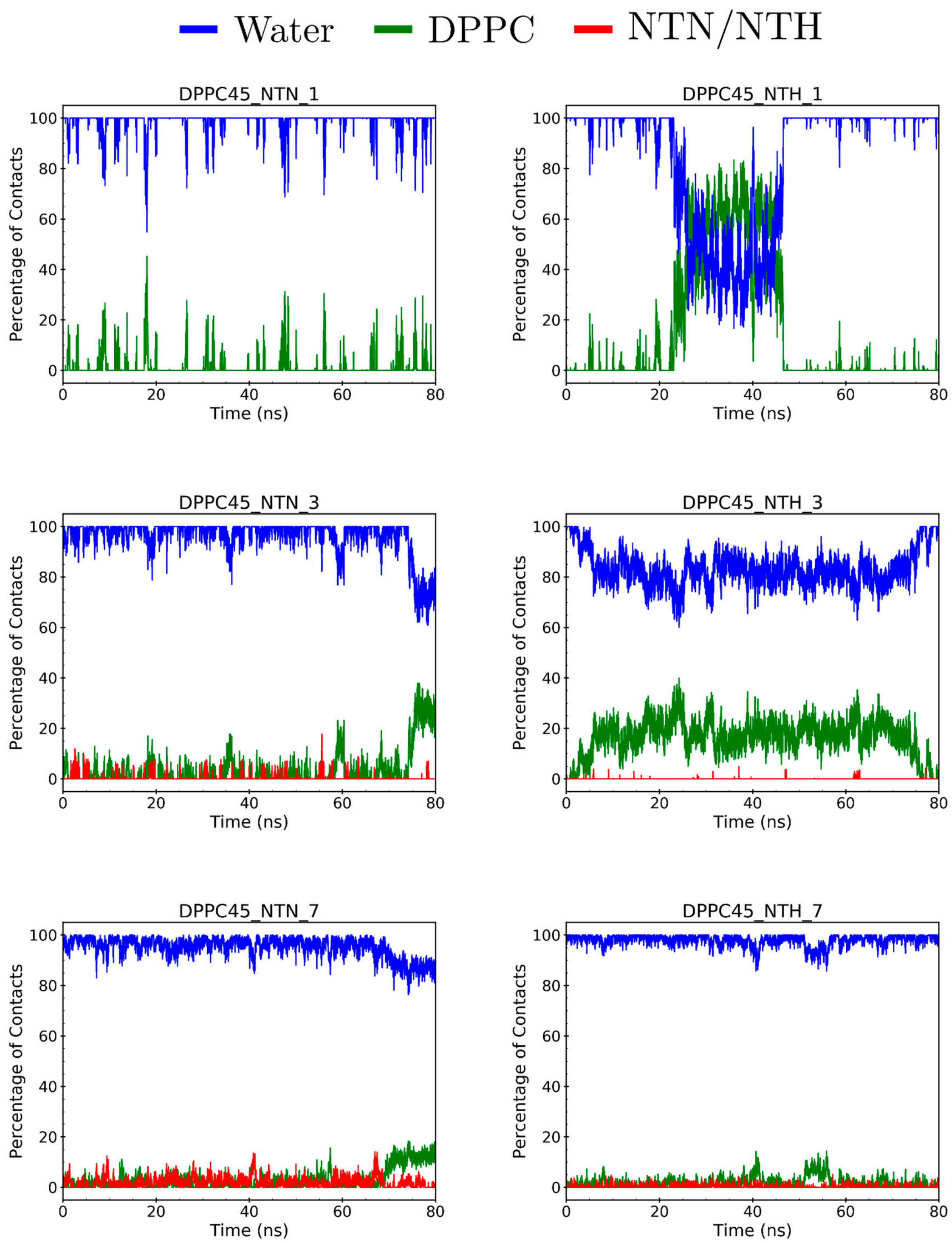

Figure S11: Time-dependent contacts, in percentage, between the protonated and neutral nicotine species with the other molecules (water, lipids, and nicotine) at different concentrations for DPPC/POPC/POPG (4:3:1 molar ratio) at 37°C. The contact is considered when the distance between any atom (including hydrogen) of two molecules is less than 3 Å. The numbers 1, 5, and 13 following the designation of each system on the top of the panels refer to the number of nicotine molecules in the system.

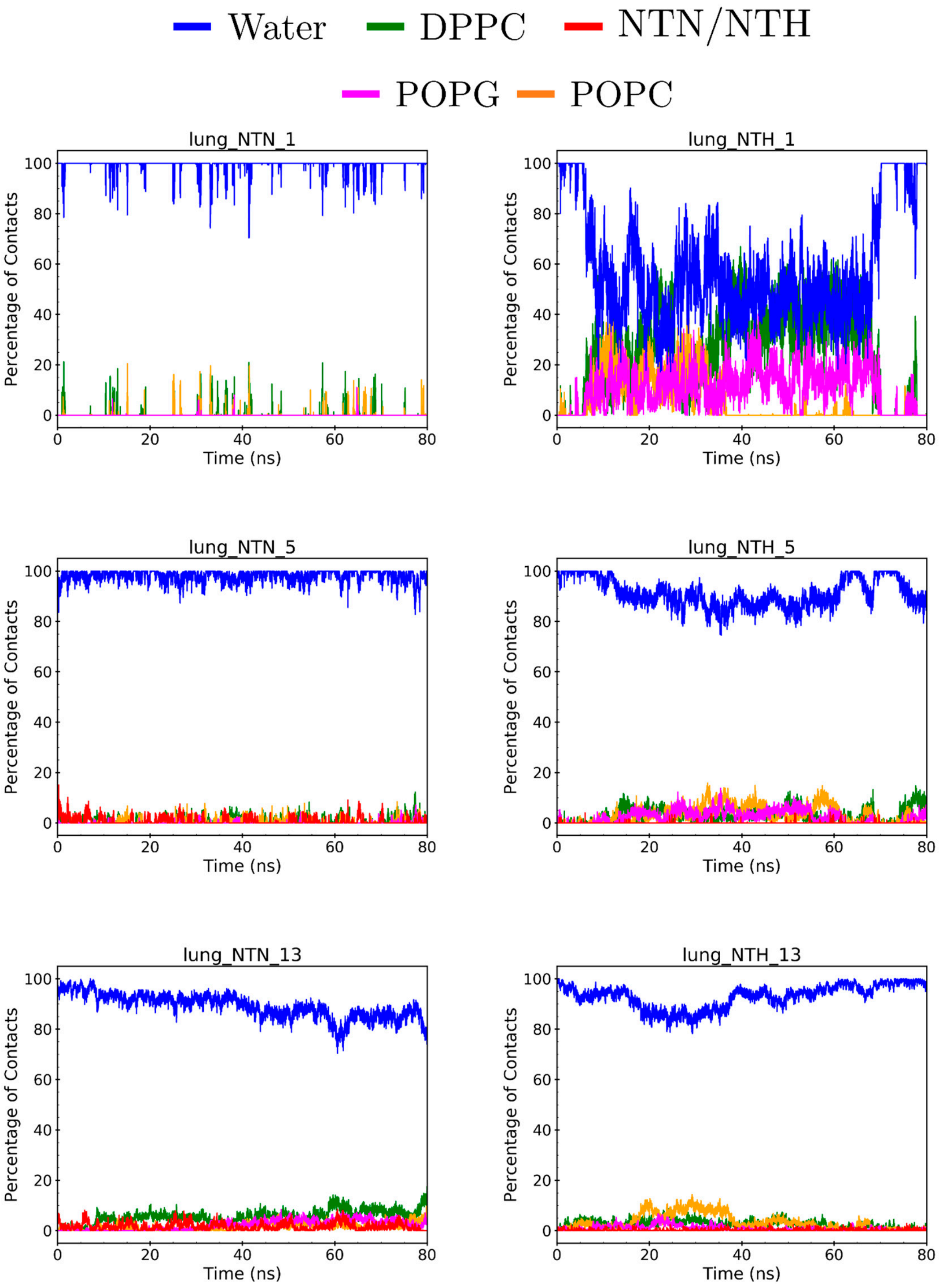

Table S1: Summary of the molecular dynamics simulation systems. The systems are identified by the membrane type (DPPC or lung), the nicotine protonation state (NTN for neutral or NTH for monoprotonated), the temperature (37 or 45 °C), and the number of nicotine molecules in the system.

System	Nicotine Protonation	Membrane Type	# of NTN or NTH	Temperature (K)
DPPC37_NTN_1	NTN	DPPC	1	310.15
DPPC37_NTN_3	NTN	DPPC	3	310.15
DPPC37_NTN_7	NTN	DPPC	7	310.15
DPPC37_NTH_1	NTH	DPPC	1	310.15
DPPC37_NTH_3	NTH	DPPC	3	310.15
DPPC37_NTH_7	NTH	DPPC	7	310.15
DPPC45_NTN_1	NTN	DPPC	1	318.15
DPPC45_NTN_3	NTN	DPPC	3	318.15
DPPC45_NTN_7	NTN	DPPC	7	318.15
DPPC45_NTH_1	NTH	DPPC	1	318.15
DPPC45_NTH_3	NTH	DPPC	3	318.15
DPPC45_NTH_7	NTH	DPPC	7	318.15
Lung_NTN_1	NTN	DPPC/POPC/POPG	1	310.15
Lung_NTN_5	NTN	DPPC/POPC/POPG	5	310.15
Lung_NTN_13	NTN	DPPC/POPC/POPG	13	310.15
Lung_NTH_1	NTH	DPPC/POPC/POPG	1	310.15
Lung_NTH_5	NTH	DPPC/POPC/POPG	5	310.15
Lung_NTH_13	NTH	DPPC/POPC/POPG	13	310.15

Table S2: Thermodynamic parameters associated with the 16-PCSL probe partitioned between the ordered and disordered phases of both DPPC and the lung surfactant model membranes at different lipid-to-drug molar ratios. The parameters were obtained by fitting the equation 2 to the experimental data up to the third order for DPPC vesicles, and to the second order for DPPC/POPC/POPG membranes.

**Sample**	**a ($x10^2$)**	**b $x10^5$ (K)**	**c $x10^7$ (K^2)**	**d $x10^{12}$ (K^3)**
DPPC				
blank	397 ± 50	-367 ± 50	1120 ± 150	-1.1 ± 0.2
25:1	155 ± 20	-142 ± 20	433 ± 30	-0.4 ± 0.1
10:1	102 ± 50	-92 ± 5	277 ± 10	-0.3 ± 0.1
DPPC/POPC/POPG				
blank	532 ± 10	-302 ± 6	42.8 ± 0.8	–
50:1	344 ± 2	-196 ± 1	27.8 ± 0.2	–
25:1	357 ± 2	-203 ± 1	28.9 ± 0.2	–
10:1	415 ± 3	-235 ± 2	33.4 ± 0.3	–

Table S3: Partial charge assigned to NTN.

Atom name	Atom type	Charge
**C**	CA_0	0.456
**H**	H4	0.106
**C1**	CA	-0.267
**C2**	CA	0.084
**H1**	HA	0.117
**C3**	CA	-0.436
**H2**	HA	0.160
**C4**	CA	0.467
**H3**	H4	0.038
**N**	NB	-0.745
**C5**	C3	0.128
**H4**	H1	0.088
**N1**	N3	-0.693
**C6**	C3	0.012
**H5**	HC	0.029
**H6**	HC	0.029
**C7**	C3	-0.196
**H7**	HC	0.058
**H8**	HC	0.058
**C8**	C3	0.164
**H9**	H1	0.059
**H10**	H1	0.059
**C9**	C3	-0.027
**H11**	H1	0.084
**H12**	H1	0.084
**H13**	H1	0.084

Table S4: Parameters assigned to NTN.

A. BONDS

Atom type	Kr	req
**CA-CA**	478.40	1.387
**CA-NB**	483.10	1.342
**CA-H4**	342.90	1.088
**C3-CA**	323.50	1.513
**CA-HA**	344.30	1.087
**C3-N3**	320.60	1.470
**C3-C3**	303.10	1.535
**C3-H1**	335.90	1.093
**C3-HC**	337.30	1092
**HT_W-HT_W**	0.00	1.514
**OT_W-HT_W**	450.00	0.957
**CA_0-CA**	478.40	1.387
**CA_0-NB**	483.10	1.342
**CA_0-H4**	342.90	1.088

B. ANGLES

Atom type	$K\Theta$	Θeq
**CA-CA-CA**	67.18	119.970
**CA-CA-C3**	63.84	120.630
**CA-NB-CA**	68.59	115.860
**CA-CA-NB**	69.16	122.630
**CA-CA-H4**	48.24	121.090
**CA-CA-HA**	48.46	120.010
**CA-C3-N3**	66.18	112.130
**CA-C3-C3**	63.25	112.090
**CA-C3-H1**	46.78	110.950
**NB-CA-H4**	51.82	115.940
**C3-N3-C3**	64.01	110.900
**C3-C3-C3**	63.21	110.630
**C3-C3-HC**	46.37	110.050
**N3-C3-C3**	66.18	110.380
**N3-C3-H1**	49.39	109.920
**C3-C3-H1**	46.36	110.070
**HC-C3-HC**	39.34	108.350
**H1-C3-H1**	39.18	109.550
**HT_W-OT_W-HT_W**	55.00	104.520
**CA_0-CA-CA**	67.18	119.970
**CA_0-CA-C3**	63.84	120.630
**CA_0-NB-CA**	68.59	115.860
**CA-CA_A-NB**	69.16	122.630
**CA-CA_0-H4**	48.24	121.090
**NB-CA_0-H4**	51.82	115.940

C. DIHEDRAL

Atom type	$k\emptyset$	n	δ
**X-CA-CA-X**	3.625	2	180.0
**X-CA-NB-X**	4.800	2	180.0
**X-C3-CA-X**	0.000	2	0.0
**X-C3-N3-X**	0.300	3	0.0
**C3-C3-N3-C3**	0.300	3	0.0
**C3-C3-N3-C3**	0.480	2	180.0
**X-C3-C3-X**	0.156	3	0.0
**C3-C3-C3-C3**	0.180	3	0.0
**C3-C3-C3-C3**	0.250	2	180.0
**C3-C3-C3-C3**	0.200	1	180.0
**HC-C3-C3-C3**	0.160	3	0.0
**HC-C3-C3-HC**	0.150	3	0.0
**CA-CA-CA_0-NB**	3.625	2	180.0
**CA-CA-CA_0-H4**	3.625	2	180.0
**NB-CA_0-CA-C3**	3.625	2	180.0
**C3-CA-CA_0-H4**	3.625	2	180.0
**CA-CA_0-NB-CA**	4.800	2	180.0
**CA-NB-CA_0-H4**	4.800	2	180.0
**CA_0-CA-CA-CA**	3.625	2	180.0
**CA_0-CA-CA-HA**	3.625	2	180.0
**CA_0-CA-C3-N3**	1.367	1	0.0
**CA_0-CA-C3-N3**	0.126	2	180.0
**CA_0-CA-C3-N3**	0.076	3	180.0
**CA_0-CA-C3-N3**	0.000	4	0.0
**CA_0-CA-C3-N3**	0.136	6	0.0
**CA_0-CA-C3-C3**	0.000	2	0.0
**CA_0-CA-C3-H1**	0.000	2	0.0
**CA_0-NB-CA-CA**	4.800	2	180.0
**CA_0-NB-CA-H4**	4.800	2	180.0

D. IMPROPERS

Atom type	K_{ω}	ω_0
**CA-H4-CA-NB**	1.100	180.0
**C3-CA-CA-CA**	1.100	180.0
**X-X-CA-HA**	1.100	180.0
**CA-H4-CA_0-NB**	1.100	180.0
**C3-CA_0-CA-CA**	1.100	180.0

E. NONBONDED

Atom type	ε	$\frac{R_{min}}{2}$	$\varepsilon\ (for\ 1-4)$	$\frac{R_{min}}{2}$ **(for 1-4)**
**CA**	-0.0860	1.9080	-0.0430	1.9080
**NB**	-0.1700	1.8240	-0.0850	1.8240
**C3**	-0.1094	1.9080	-0.0547	1.9080
**N3**	-0.1700	1.8240	-0.0850	1.8240
**H4**	-0.0150	1.4090	-0.0075	1.4090

**HA**	-0.0150	1.4590	-0.0075	1.4590
**H1**	-0.0157	1.3870	-0.0078	1.3870
**HC**	-0.0157	1.4870	-0.0078	1.4870
**HT_W**	-0.0460	0.2245		
**OT_W**	-0.1521	1.7685		
**CA_0**	-0.0860	1.9080	-0.0430	1.9080

Table S5: Partial charge assigned to NTH.

Atom name	Atom type	Charge
**C**	CA_0	0.271
**H**	H4	0.064
**C1**	CA	-0.186
**C2**	CA	0.020
**H1**	HA	0.142
**C3**	CA	-0.312
**H2**	HA	0.176
**C4**	CA	0.339
**H3**	H4	0.081
**N**	NB	-0.513
**C5**	C3	0.119
**H4**	HX	0.102
**N1**	N4	-0.258
**H5**	HN	0.325
**C6**	C3	-0.129
**H6**	HC	0.090
**H7**	HC	0.090
**C7**	C3	-0.078
**H8**	HC	0.077
**H9**	HC	0.077
**C8**	C3	0.064
**H10**	HX	0.086
**H11**	HX	0.086
**C9**	C3	-0.045
**H12**	HX	0.104
**H13**	HX	0.104
**H14**	HX	0.104

Table S6: Parameters assigned to NTH.

A. BONDS

Atom type	Kr	req
**CA-CA**	478.40	1.387
**CA-NB**	483.10	1.342
**CA-H4**	342.90	1.088
**C3-CA**	323.50	1.513
**CA-HA**	344.30	1.087
**C3-N4**	293.60	1.499
**C3-C3**	303.10	1.535
**C3-HX**	338.70	1.091
**HN-N4**	369.00	1.033
**C3-HC**	337.30	1.092
**HT_W-HT_W**	0.00	1.514
**OT_W-HT_W**	450.00	0.957
**CA_0-CA**	478.40	1.387
**CA_0-NB**	483.10	1.342
**CA_0-H4**	342.90	1.088

B. ANGLES

Atom type	$K\Theta$	Θeq
**CA-CA-CA**	67.18	119.970
**CA-CA-C3**	63.84	120.630
**CA-NB-CA**	68.59	115.860
**CA-CA-NB**	69.16	122.630
**CA-CA-H4**	48.24	121.090
**CA-CA-HA**	48.46	120.010
**CA-C3-N4**	64.87	114.540
**CA-C3-C3**	63.25	112.090
**CA-C3-HX**	46.69	111.440
**NB-CA-H4**	51.82	115.940
**C3-N4-C3**	62.84	110.640
**C3-N4-HN**	46.19	110.110
**C3-C3-C3**	63.21	110.630
**C3-C3-HC**	46.37	110.050
**N4-C3-C3**	64.45	114.320
**N4-C3-HX**	49.02	107.910
**C3-C3-HX**	46.02	111.740
**HC-C3-HC**	39.34	108.350
**HX-C3-HX**	39.04	110.740
**HT_W-OT_W-HT_W**	55.00	104.520
**CA_0-CA-CA**	67.18	119.970
**CA_0-CA-C3**	63.84	120.630
**CA_0-NB-CA**	68.59	115.860
**CA-CA_A-NB**	69.16	122.630
**CA-CA_0-H4**	48.24	121.090
**NB-CA_0-H4**	51.82	115.940

C. DIHEDRAL

Atom type	$k\emptyset$	n	δ
**X-CA-CA-X**	3.625	2	180.0
**X-CA-NB-X**	4.800	2	180.0
**X-C3-CA-X**	0.000	2	0.0
**X-C3-N4-X**	0.156	3	0.0
**X-C3-C3-X**	0.156	3	0.0
**C3-C3-C3-C3**	0.180	3	0.0
**C3-C3-C3-C3**	0.250	2	180.0
**C3-C3-C3-C3**	0.200	1	180.0
**HC-C3-C3-C3**	0.160	3	0.0
**HC-C3-C3-HC**	0.150	3	0.0
**CA-CA-CA_0-NB**	3.625	2	180.0
**CA-CA-CA_0-H4**	3.625	2	180.0
**NB-CA_0-CA-C3**	3.625	2	180.0
**C3-CA-CA_0-H4**	3.625	2	180.0
**CA-CA_0-NB-CA**	4.800	2	180.0
**CA-NB-CA_0-H4**	4.800	2	180.0
**CA_0-CA-CA-CA**	3.625	2	180.0
**CA_0-CA-CA-HA**	3.625	2	180.0
**CA_0-CA-C3-N4**	0.482	1	0.0
**CA_0-CA-C3-N4**	0.778	2	0.0
**CA_0-CA-C3-N4**	0.143	3	180.0
**CA_0-CA-C3-N4**	0.260	4	0.0
**CA_0-CA-C3-N4**	0.273	6	0.0
**CA_0-CA-C3-C3**	0.000	2	0.0
**CA_0-CA-C3-HX**	0.000	2	0.0
**CA_0-NB-CA-CA**	4.800	2	180.0
**CA_0-NB-CA-H4**	4.800	2	180.0

D. IMPROPERS

Atom type	K_ω	ω_0
**CA-H4-CA-NB**	1.100	180.0
**C3-CA-CA-CA**	1.100	180.0
**X-X-CA-HA**	1.100	180.0
**CA-H4-CA_0-NB**	1.100	180.0
**C3-CA_0-CA-CA**	1.100	180.0

E. NONBONDED

Atom type	ε	$\frac{R_{min}}{2}$	$\varepsilon\ (for\ 1-4)$	$\frac{R_{min}}{2}$ **(for 1-4)**
**CA**	-0.0860	1.9080	-0.0430	1.9080
**NB**	-0.1700	1.8240	-0.0850	1.8240
**C3**	-0.1094	1.9080	-0.0547	1.9080
**N3**	-0.1700	1.8240	-0.0850	1.8240
**H4**	-0.0150	1.4090	-0.0075	1.4090
**HA**	-0.0150	1.4590	-0.0075	1.4590
**HX**	-0.0157	1.1000	-0.0078	1.1000

**HN**	-0.0157	0.6000	-0.0078	0.6000
**HC**	-0.0157	1.4870	-0.0078	1.4870
**HT_W**	-0.0460	0.2245		
**OT_W**	-0.1521	1.7685		
**CA_0**	-0.0860	1.9080	-0.0430	1.9080

References

1. Basso, L.G.M.; Rodrigues, R.Z.; Naal, R.M.Z.G.; Costa-Filho, A.J. Effects of the Antimalarial Drug Primaquine on the Dynamic Structure of Lipid Model Membranes. *Biochim Biophys Acta Biomembr* **2011**, *1808*, 55–64, doi:10.1016/j.bbamem.2010.08.009.

2. Clayton, P.M.; Vas, C.A.; Bui, T.T.T.; Drake, A.F.; McAdam, K. Spectroscopic Investigations into the Acid–Base Properties of Nicotine at Different Temperatures. *Analytical Methods* **2012**, *5*, 81–88, doi:10.1039/C2AY25678A.
